# The burden of premature ventricular contractions predicts adverse fetal and neonatal outcomes among pregnant women without structural heart disease: A prospective cohort study

**DOI:** 10.1002/clc.23612

**Published:** 2021-05-06

**Authors:** Jing Lin, Yanxia Qian, Qiushi Chen, Mingming Zhang, Yaoxi Chen, Ruijie Xu, Jingxian Chen, Yukang Shi, Shunxin Yang, Xinyi Luo, Qiang Ding, Xin Wu, Junhong Wang

**Affiliations:** ^1^ Department of Cardiology The First Affiliated Hospital with Nanjing Medical University Nanjing Jiangsu China; ^2^ Department of Medicine Nanjing Medical University Nanjing Jiangsu China; ^3^ Department of Breast Surgery The First Affiliated Hospital with Nanjing Medical University Nanjing Jiangsu China; ^4^ Department of Obstetrics The First Affiliated Hospital with Nanjing Medical University Nanjing Jiangsu China; ^5^ Department of Cardiology Xinjiang Yili Friendship Hospital, Yili Kazak Autonomous Prefecture Xinjiang China

**Keywords:** fetal/neonatal outcomes, pregnancy, premature ventricular contractions (PVCs)

## Abstract

**Background:**

Premature ventricular contractions (PVCs) may increase during pregnancy, however, few studies have evaluated the relationship between PVCs and the pregnant outcomes.

**Hypothesis:**

PVCs may increase the adverse fetal/neonatal outcomes in pregnant women.

**Methods:**

Six thousand one hundred and forty‐eight pregnant women were prospectively enrolled in our center between 2017 and 2019 in the study. The average PVC burden was determined by calculating the number of PVCs in total beats. Those who had a PVC burden >0.5% were divided into two groups based on the presence or absence of adverse fetal or neonatal events. The adverse outcomes were compared between the groups to assess the impact of PVCs on pregnancy.

**Results:**

A total of 103 (1.68%) women with a PVC burden >0.5% were recorded. Among them, 17 adverse events (12 cases) were documented, which was significantly higher than that among women without PVCs (11.65% vs. 2.93%, p < .01). The median PVC burden among pregnant women with PVCs was 2.84% (1.02%–6.1%). Furthermore, compared with that of the women without adverse events, the median PVC burden of women with adverse fetal or neonatal outcomes was significantly higher (9.02% vs. 2.30%, p < .01). Multivariate logistic regression analysis demonstrated that not the LVEF, heart rate and bigeminy, but only the PVC burden was associated with adverse fetal or neonatal outcomes among pregnant women with PVCs (OR: 1.34, 95% CI [1.11–1.61], p < .01).

**Conclusions:**

Frequent PVCs have adverse effects on pregnancy, and the PVC burden might be an important factor associated with adverse fetal and neonatal outcomes among pregnant women with PVCs.

## 1 INTRODUCTION

Idiopathic premature ventricular contractions (PVCs) are relatively benign in cases without structural heart diseases but may signal an increased risk of sudden death in cases with structural heart disease (SHD) and may be markers of underlying pathology. An estimated prevalence of 1%–4% is found in the general population on standard 12‐lead electrocardiography (ECG).^1^ Frequent PVCs, defined as more than 10% of all QRS complexes on standard 24 h Holter monitoring, have been found to be associated with subsequent development of left ventricular dilatation and PVC‐induced cardiomyopathy. Treatment of frequent PVCs in patients with impaired ventricular function can reverse this pattern.^2^, ^3^, ^4^, ^5^, ^6^, ^7^ PVCs may increase during pregnancy, which leads to an additional challenge, as pregnancy outcome implications are not known for this already cardiac overloaded state.^8^, ^9^ Relatively few studies have evaluated the relationship between PVCs and the adverse outcomes of fetuses or neonates.^10^ Therefore, we aimed to determine the relationship between the adverse outcomes of the fetus and neonate among pregnant women with PVCs.

## 2 METHODS

### 2.1 Study design and patient population

This is a single‐center prospective cohort study of consecutive pregnancies referred to The First Affiliated Hospital of Nanjing Medical University from July 2017 to July 2019. The inclusion criteria were pregnant women with normal cardiac structure and function based on echocardiographic examination. Pregnant women with PVC burdens greater than 0.5% on Holter examination each time during the whole pregnancy period were classified as the PVC group. The exclusion criteria were as follows: Pregnant women with (1) hyperthyroidism, (2) hypothyroidism, (3) gestational hypertension or pregnancy with hypertension, (4) Type I or II diabetes, (5) chronic nephritis or impaired renal function (creatinine clearance rate, Ccr <60 ml/min), (6) autoimmune disease, (7) congenital heart diseases, (8) pulmonary artery hypertension, (9) cardiomyopathy, (10) alcohol abuse, (11) anemia (Hgb <110 g/L), (12) smoking, and (13) a family history of sudden cardiac death. The study protocol was performed in accordance with the ethical standards established in the 1964 Declaration of Helsinki and its later amendments and was approved by the institutional ethics committee of The First Affiliated Hospital of Nanjing Medical University (2019SR‐503). Informed consent was obtained from all pregnant women enrolled in the study.

### 2.2 Baseline characteristics

Clinical and 12‐lead ECG data were collected at the time of the initial clinic visit or for routine obstetric examinations during the first trimester of pregnancy. If the standard 12‐lead ECG recorded the PVC events, Holter monitoring was then performed and reexamined in each subsequent trimester to assess the burden of ventricular premature contractions and analyze the total number of QRS complexes, total number of PVCs and total runs of nonsustained ventricular tachycardia (NSVT) (run with >3 consecutive PVCs but <30 s). Furthermore, if the women had self‐reported symptoms such as palpitation during pregnancy, the 12‐lead ECG examination was then performed. If no arrhythmia was recorded, a wearable single‐lead ECG recorder (Shuweikang company, Nanjing, China) was then weared for three continuous days to record the possible PVC events in those pregnant women. The single‐lead wearable ECG recorder was designed to recognize the arrhythmia based on the convolutional neural network technology. It can easily identify the PVCs when it happens. And if the PVCs was documented by 12‐lead ECG or the wearable single‐lead ECG recorder and confirmed by the ECG experts in our center (Lin J & Chen QS), the Holter was examined thereafter once a trimester for the remainder of the pregnancy period. The average PVC burden was determined by calculating the total number of PVCs in the total QRS complexes. PVC morphological patterns were analyzed to determine the PVC origin. PVCs with inferior axis and left bundle branch block (LBBB) patterns were determined to most likely originate from the right ventricular outflow tract (RVOT). Specific diagnostic procedures were carried out with reference to published diagnostic criteria.^11^ Specifically, the burden, morphology and the origin of PVCs were calculated not by the wearable recorder but by the Holter and 12‐lead ECG results due to its single lead characteristics.

Echocardiography was performed in the first and third trimesters in all pregnant women. Experienced echocardiographers performed echocardiography to ensure that there was no evidence of SHD in the pregnant women and to ensure that the cardiac function of the enrolled pregnant women was within the normal range (left ventricular ejection function [LVEF] >60%).

All pregnant women were followed, and the data on characteristics including age, comorbid medical conditions and family history were recorded. All women were followed from preconception to 1 week after delivery. If the exclusion criteria were met, they were excluded from the study.

### 2.3 Neonatal/fetal outcome events

Two physicians blinded to the women's baseline characteristics independently verified adverse events. Adverse fetal and neonatal events were defined as previously described^10^: Premature birth (<37 weeks gestation), small‐for‐gestational‐age birth weight (<10th percentile for gestational age or < 2500 g), respiratory distress syndrome, intraventricular hemorrhage and fetal death (after 20 weeks gestation and before birth). Only one event was counted if multiple events occurred in the fetus or newborn simultaneously.

### 2.4 Statistical analysis

Statistical analysis was performed using the SPSS (Version 22.0). Data are presented as the means ± SDs, medians (25th–75th percentiles), or proportions. Student's *t*‐test or the Wilcoxon rank‐sum test was used to compare continuous variables between the women with and without adverse fetal/neonatal events who had PVCs >0.5%. Possible risk factors of adverse events were analyzed using univariable logistic regression, followed by multivariable logistic regression. A p value <.05 was considered statistically significant.

## 3 RESULTS

### 3.1 Baseline characteristics and fetal or neonatal outcomes

**FIGURE 1:**
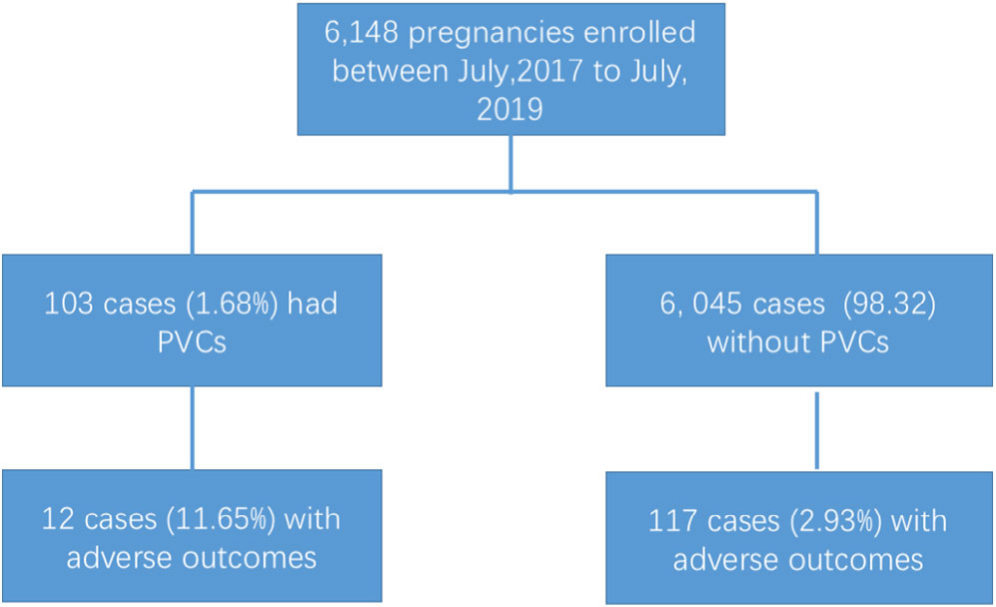
Summarized flow chart for the study findings and the fetal/neonatal outcomes of pregnancies

**FIGURE 2:**
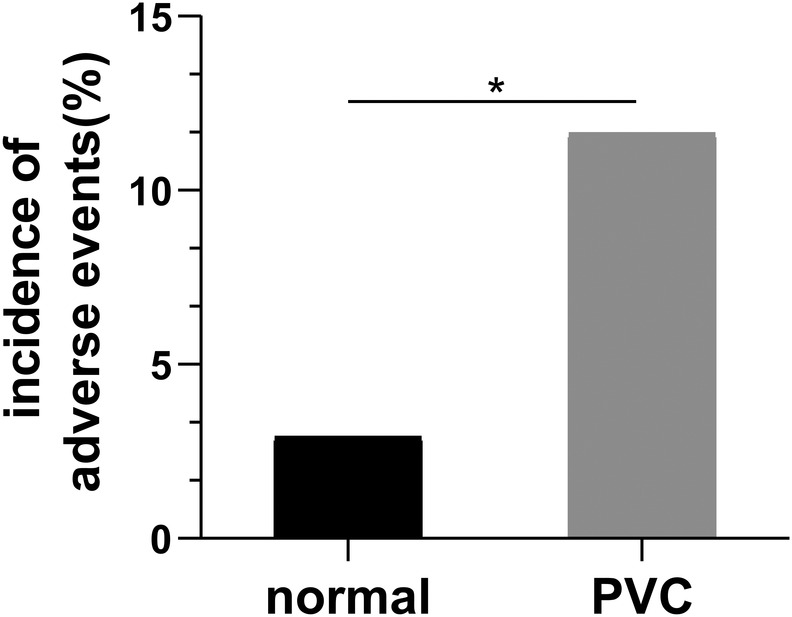
The incidence of adverse events in pregnant women with or without premature ventricular contraction (PVC). The incidence of adverse events is higher in PVC cases (11.65%) than normal controls without PVC (2.93%). * indicates p < .05

Six thousand one hundred and forty‐eight pregnant women who fulfilled the inclusion criteria were enrolled consecutively, of which 103 (1.68%) had symptomatic or asymptomatic PVCs. A total of 17 adverse events occurred in 103 pregnant women with PVCs, including five cases of respiratory distress syndrome, five preterm births, and seven small‐for‐gestational‐age births. Finally, only 12 women with adverse events were counted, as multiple events occurred in some of them simultaneously. The remaining defined adverse events did not occur. The other 91 pregnant women with PVCs delivered safely without adverse events. A total of 177 fetal and neonatal adverse cases were counted in the cohort of 6045 pregnant women without PVCs. The incidence of adverse events was significantly higher in PVC cases (11.65%) than in those without PVC (2.93%) (Figures 1 and 2).

### 3.2 Cardiac rhythm of pregnant women with PVCs

The pregnant women with PVCs were divided into two groups on the basis of the presence or absence of adverse events. The baseline characteristics are listed in Table 1. The proportion of bigeminy PVCs was significantly higher in pregnant women with adverse fetal or neonatal outcomes (50% vs. 19.8%, p < .05), as was the median PVC burden, than in women without adverse events (9.02% vs. 2.30%, p < .01). The median documented PVC burden was 2.84% (1.02%–6.10%). Eight pregnancies had a maximal PVC burden >10%, and of these, two pregnancies had a PVC burden >20%. Although the LVEF of the adverse outcome group was within the normal range, it remained slightly lower than that of the control group (64.16% ± 1.56% vs. 65.69% ± 2.54%, p < .05). There were no other significant differences in the remaining baseline data between the two groups.

**TABLE 1:** Baseline characteristics of pregnant women with premature ventricular contractions

Characteristic	No adverse events (*n* = 91)	Adverse events (*n* = 12)	p value
Age, years	30.46 ± 4.62	32.67 ± 5.55	.132
Previous abortion(%)	32(35.2)	5(41.7)	.752
Hgb, g/L	121.40 ± 9.52	121.00 ± 8.54	.892
HR, bpm	85.93 ± 9.65	92.08 ± 6.52	.035
ECG			
V‐Bigeminy(%)	18(19.8)	6(50.0)	.030
Multifocal PVC(%)	2(2.2)	2(16.7)	.066
NSVT(%)	1(1.1)	0(0)	1.000
Origin			
LV(%)	28(30.8)	5(41.7)	.515
PVC burden(%)	2.30(0.88,4.85)	9.02(7.43,11.71)	.000
LVEF	65.69 ± 2.54	64.16 ± 1.56	.032
Childbirth type			
Natural childbirth(%)	55(60.4)	5(41.7)	.351

*Note*: Data are expressed as mean ± SD, medians (25th–75th percentiles), or number (percentage).

Abbreviations: ECG, electrocardiogram; Hgb, hemoglobin; HR, heart rate; LV, left ventricular; LVEF, left ventricular ejection fraction; NSVT, non‐sustained ventricular tachycardia; PVC, premature ventricular contraction.

Twelve‐lead ECG showed normal sinus rhythm with premature ventricular complexes in the study. PVCs with an LBBB and inferior axis, most likely originating from RVOT, were found in 41.75% of pregnancies with PVCs. In contrast, PVCs with an Right Bundle Branch Block (RBBB) and inferior axis, frequently associated with the left ventricular outflow tract (LVOT), accounted for approximately 11.65%, far less than PVCs associated with the RVOT. According to the special morphology of PVCs reported in the previous literature,^12^, ^13^, ^14^, ^15^ PVCs originating from the tricuspid annulus account for approximately 12.62% and 20.39% of PVCs originating from the papillary muscles or fascicle of the left ventricle, respectively. A total of 9.71% of PVC origins could not be determined from the ECG (Figure S1).

### 3.3 Predictors of fetal and neonatal outcomes

**FIGURE 3:**
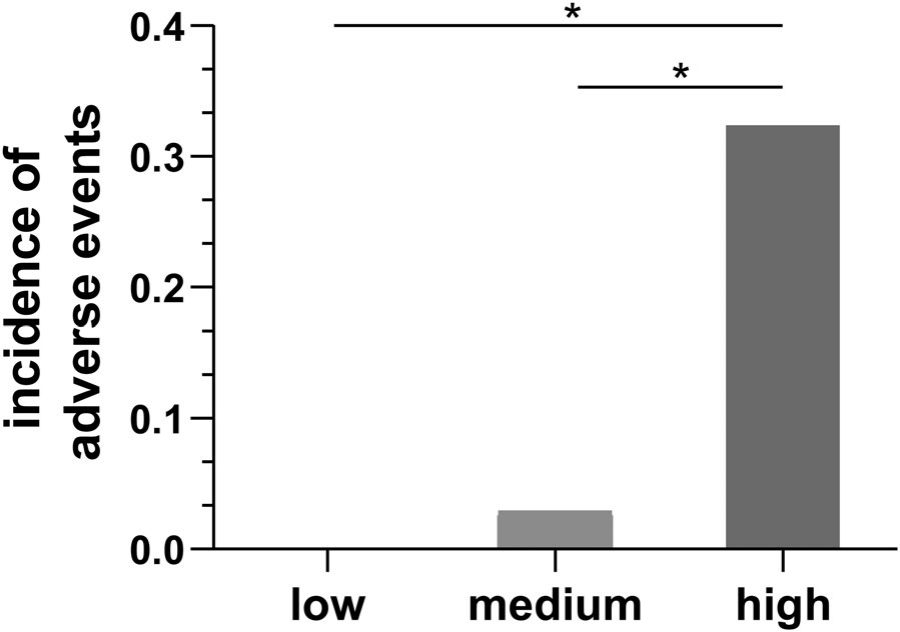
Incidence of adverse fetal events in low premature ventricular contraction (PVC) burden (low), medium PVC burden (medium) and high PVC burden group (high). low PVC burden: < The 33rd percentile (<1.69%), medium PVC burden: The 33rd percentile–the 67th percentile (1.69%–4.77%), high PVC burden: > The 67th percentile (>4.77%), * indicates p < .05

The baseline characteristics of the study cohort suggested that the average burden of PVCs was related to adverse outcomes, so the burden of PVCs was further divided into low (<33rd percentile), middle (33rd–67th percentile) and high groups (>67th percentile) according to the percentile of the average PVC burden (Figure 3). The incidence of adverse events was significantly higher in the high‐burden PVC group than in the low and middle group.

Univariable logistic regression analysis demonstrated that the LVEF, heart rate, bigeminy and burden of PVCs were associated with adverse fetal or neonatal outcomes among pregnant women with PVCs. Variables which were statistically significant in univariate analysis were further analyzed in multivariate logistic regression analysis; however, statistical significance was only evident for PVC burden in the multivariate logistic regression analysis (OR: 1.34, 95% CI [1.11–1.61], p < .05, Table 2).

**TABLE 2:** Logistic regression analysis for adverse events in pregnant women with PVCs

	Univariable analysis OR (95% CI)			Multivariable analysis OR (95% CI)		
	OR	95%CI	p value	OR	95%CI	p value
V‐bigeminy	4.06	(1.17,14.07)	.027			
PVC burden	1.35	(1.14,1.58)	.000	1.30	(1.08,1.57)	.005
HR	1.08	(1.00,1.16)	.040			
LVEF	0.76	(0.58,1.00)	.049			

Abbreviations: CI, confidence interval; HR, heart rate; LVEF, left ventricular ejection fraction; OR, odds ratio; PVC, premature ventricular contraction.

## 4 DISCUSSION

It is widely recognized that idiopathic PVCs are relatively benign in structurally normal hearts;^16^ however, relatively few studies have evaluated the relationship between PVCs and the adverse outcomes of fetuses or neonates among pregnant women.^10^, ^17^ In this study, we found that PVCs were associated with a higher frequency fetal/neonatal adverse events among pregnant women with a structurally normal heart. In fact, we further demonstrated that 'high' PVC burden is an important factor for predicting the incidence of adverse fetal/neonatal outcomes.

In our study, 1.68% pregnancies with structurally normal heart were found to have high PVC burden in 6148 consecutive enrolled pregnant women. And the rate of adverse fetal/neonatal events in women with PVCs was 11.65%. Similar results were reported in Tong C et al.,^10^ who enrolled 53 consecutive pregnancies with PVCs and found 13% adverse fetal and/or neonatal events. However, in the group of women without PVCs, the incidence rate was only 2.93% in our study, which was significantly lower than that previously reported in the group of pregnant women with or without structural heart disease.^10^, ^18^ The reasons might be that comorbidities that may cause adverse fetal/neonatal outcomes, such as diabetes, hypertension or hyperthyroidism, were excluded from our study. In fact, when the pregnant women with PVCs were divided by outcomes, a significantly high PVC burden, a high rate of bigeminal PVCs and slightly decreased LVEF were associated with a high incidence of fetal/neonatal events. Further multivariate logistic analysis suggested that a high PVC burden was the only predictor of adverse fetal/neonatal outcomes among women with PVCs. In contrast to previous reports,^10^ our study revealed a close relationship between PVCs and adverse fetal/neonatal outcomes among pregnant women, suggesting that more active medication or even invasive strategies such as radiofrequency ablation should be considered for women with a high PVCburden (PVCs burden>4.77%) before or during pregnancy.^19^ Furthermore, it is undeniable to say that some of pregnant women with PVCs would not be captured through an intermittent screening 12‐lead EKG, which may cause misclassification of women with PVCs into those without PVCs. We, therefore, use the wearable single‐lead ECG recorder to identify the possible PVC in those women with self‐reported symptoms such as palpitation to reduce the possible misclassification.

Idiopathic PVCs are distributed mainly to specific cardiac structural sites, of which the RVOT accounts for approximately 67%, and the rest are mainly followed by the LVOT around the annulus, papillary muscles and other special sites.^20^ The distribution of the origin of PVCs in all pregnant women was documented to investigate the relationship between the origin of PVCs and adverse pregnancy outcomes. Unlike the studies by Nakagawa et al.^9^ and Tong et al.,^10^ which reported that 73%–92% of PVCs originated from RVOT in pregnant women, we found that approximately 57.28% of PVCs originated from the right ventricle, of which only 41.75% originated from the RVOT. In addition, PVCs of the left ventricle accounted for approximately 32.04% in our study, which was significantly higher than that in the normal population. The possible mechanism may be the reason that the left ventricle is more sensitive to volumetric load than the right ventricle. However, because the origin of 9.71% of PVCs could not be identified based on the 12‐lead ECG in our study, this may have an impact on classification of the true distribution of PVCs in pregnant women.

### 4.1 Study limitations

Although this study is a prospective, single‐center study, the number of participants included is still relatively small; therefore, a large‐scale and multicenter study should be performed in the future. Second, PVC origin was classified by a standard 12‐lead ECG pattern in the study. Undeniably, changes in diaphragm position during pregnancy and cardiac rotation may cause misjudgment of the origin of PVCs. Third, because the PVC burden may fluctuate throughout the day and throughout stages of pregnancy, we applied the average PVC burden screened by Holter in each trimester to represent the PVC burden during pregnancy. It is therefore suggested to use long‐term wearable monitoring equipment to screen the PVC burden in the future. Finally, all patients underwent only two‐dimensional echocardiography in the study, and cardiac magnetic resonance might be suitable for further study to eliminate the possibility of latent cardiomyopathy.

In conclusion, our study revealed that the prevalence of PVCs in pregnancy is higher than that in the normal population. Frequent PVCs have adverse effects on pregnancy, and the PVC burden might be the main factor associated with adverse pregnancy outcomes.

## CONFLICT OF INTEREST

The authors declare no potential conflict of interest.

## AUTHOR CONTRIBUTIONS

Junhong Wang, Xin Wu: Designed, supervised the study and prepared the manuscript. Jing Lin, Yanxia Qian, Qiushi Chen: Data collection and analysis, writing original draft. Yaoxi Chen, Ruijie Xu, Jingxian Chen, Yukang Shi, Shunxin Yang, and Xinyi Luo: Data collection and analysis. Mingming Zhang: Writing ‐ Review and Editing. Qiang Ding: Project administration and data analysis.

## Supporting information


**Figure S1** The origin of PVC in pregnant women. LVOT, left ventricular outflow tract; RVOT, right ventricular outflow tract; TV, tricuspid valve; LP, left posterior; LA, left anterior; others indicate 10 unidentified classifications, 1 from right ventricular inflow tract, 1 from right ventricular apex, 1 from His bundle and 1 from right ventricular mid‐septum.

## Data Availability

The data that support the findings of this study are openly available in DOI: https://doi.org/10.17632/7s38jdjnf9.2.
