# Supplementary figures and images for: The burden of premature ventricular contractions predicts adverse fetal and neonatal outcomes among pregnant women without structural heart disease: A prospective cohort study
Source: Clin Cardiol. 2021 May 6;44(6):833–8. doi: 10.1002/clc.23612 (PMC8207974; doi:10.1002/clc.23612)

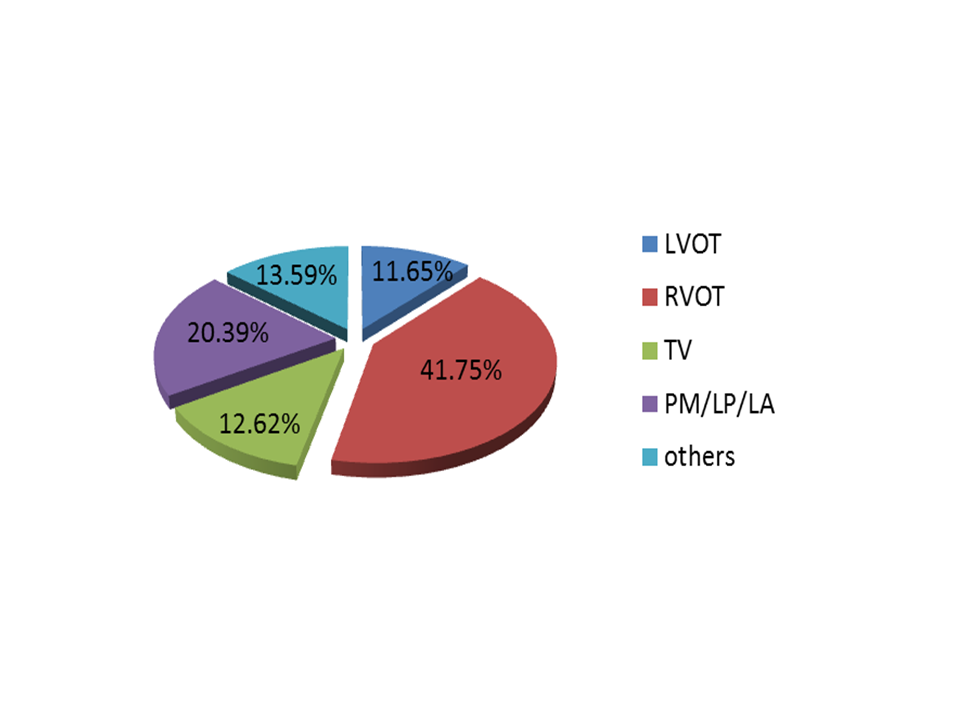

Supplement: Supplementary file 1 — Figure S1 The origin of PVC in pregnant women. LVOT, left ventricular outflow tract; RVOT, right ventricular outflow tract; TV, tricuspid valve; LP, left posterior; LA, left anterior; others indicate 10 unidentified classifications, 1 from right ventricular inflow tract, 1 from right ventricular apex, 1 from His bundle and 1 from right ventricular mid‐septum. [file CLC-44-833-s001.tif]
